# Analysis of genotyping data reveals the unique genetic diversity represented by the breeds of sheep native to the United Kingdom

**DOI:** 10.1186/s12863-024-01265-3

**Published:** 2024-09-17

**Authors:** Eleanor Kerr, Melissa M. Marr, Lauren Collins, Katie Dubarry, Mazdak Salavati, Alissa Scinto, Shernae Woolley, Emily L. Clark

**Affiliations:** 1 The Roslin Institute, University of Edinburgh, Easter Bush Campus, Easter Bush, Midlothian, EH25 9RG United Kingdom; 2 Dairy Research Centre, Scotland’s Rural College (SRUC), Barony Campus, Dumfries, DG1 3NE United Kingdom

**Keywords:** *Ovis aries*, SNP genotyping, Genetic diversity, Breed conservation, Population structure, Admixture

## Abstract

**Background:**

Sheep breeds native to the United Kingdom exhibit a striking diversity of different traits. Some of these traits are highly sustainable, such as seasonal wool shedding in the Wiltshire Horn, and are likely to become more important as pressures on sheep production increase in coming decades. Despite their clear importance to the future of sheep farming, the genetic diversity of native UK sheep breeds is poorly characterised. This increases the risk of losing the ability to select for breed-specific traits from native breeds that might be important to the UK sheep sector in the future. Here, we use 50 K genotyping to perform preliminary analysis of breed relationships and genetic diversity within native UK sheep breeds, as a first step towards a comprehensive characterisation. This study generates novel data for thirteen native UK breeds, including six on the UK Breeds at Risk (BAR) list, and utilises existing data from the publicly available Sheep HapMap dataset to investigate population structure, heterozygosity and admixture.

**Results:**

In this study the commercial breeds exhibited high levels of admixture, weaker population structure and had higher heterozygosity compared to the other native breeds, which generally tend to be more distinct, less admixed, and have lower genetic diversity and higher kinship coefficients. Some breeds including the Wiltshire Horn, Lincoln Longwool and Ryeland showed very little admixture at all, indicating a high level of breed integrity but potentially low genetic diversity. Population structure and admixture were strongly influenced by sample size and sample provenance – highlighting the need for equal sample sizes, sufficient numbers of individuals per breed, and sampling across multiple flocks. The genetic profiles both within and between breeds were highly complex for UK sheep, reflecting the complexity in the demographic history of these breeds.

**Conclusion:**

Our results highlight the utility of genotyping data for investigating breed diversity and genetic structure. They also suggest that routine generation of genotyping data would be very useful in informing conservation strategies for rare and declining breeds with small population sizes. We conclude that generating genetic resources for the sheep breeds that are native to the UK will help preserve the considerable genetic diversity represented by these breeds, and safe-guard this diversity as a valuable resource for the UK sheep sector to utilise in the face of future challenges.

**Supplementary Information:**

The online version contains supplementary material available at 10.1186/s12863-024-01265-3.

## Background

In the UK, the sheep (*Ovis aries L. 1758*) industry is a longstanding and vital agricultural sector. In 2020, it employed approximately 150,000 individuals and contributed £290 million to the UK economy [1]. Some of the sheep breeds, such as the Suffolk, that are native to the UK have become a mainstay of sheep production across the globe [2]. Others such as the Longwool breeds have had a significant historical effect on breed formation across the globe [3]. Despite their importance to global sheep production, the unique genetic composition of native UK sheep breeds is poorly characterised; large-scale genomic studies lack the resolution to examine large numbers of local breeds [4, 5] and, until recently [2], they have been underrepresented in the genetic and genomic resources that are available for sheep breeds globally [6].

Maintenance of genetic diversity in native UK sheep breeds is essential for the conservation of desirable traits, such as those that might be important for mitigating future health and/or climatic challenges, as well as the long-term survival of breeds (inbreeding risk) with small and declining populations [7]. Globally, breed diversity within livestock species is in decline [8, 9]. The promotion of a few high-yielding types for intensive production has led to the dominance of a small number of production breeds and the consequent decline of locally adapted, regional breeds. Predicted changes in climate and pressure from emerging and existing diseases are likely to significantly impact livestock, including sheep [10–12]. Resilience to these emerging pressures is linked to long-term maintenance of genetic diversity [8, 9]. Loss of these breeds, and/or loss of within-breed genetic diversity, would remove important genetic resources that are likely to become important to the UK sheep sector in the future, posing a threat to future food security and breed conservation.

Several native breeds of sheep possess traits that are desirable in changing and unpredictable climates [1, 13, 14]. The Norfolk Horn is particularly hardy and can adapt easily to the hot dry summers and long wet winters we are likely to experience in coming years as a consequence of climate change [14]. The Dorset Horn is able to reproduce a-seasonally which can also enhance the resilience of production systems to fluctuating weather patterns [14]. The Lincoln Longwool is also deemed hardy with increased resistance to footrot [14]. Footrot is a painful infection affecting sheep hooves that can result in lameness and is a cause of economic losses in sheep production globally [15]. It is particularly common in warm, wet conditions [16] and could become more of a problem if predicted changes to climate in the UK become reality [17]. In addition, there may be traits that have not yet been discovered that are useful within the UK’s native breeds. For example, it has previously been found that some sheep breeds exhibit heightened susceptibility to specific infections or suffer more severe cases [18], and benefit from cross-breeding with others breeds that are either resistant or less susceptible. Genetic diversity is a frontline defence against potential disease pressures. If the genetic diversity represented by the UK native breeds is lost, we also lose the option to exploit it for the purposes of mitigating future disease challenges. As such preserving genetic diversity is vital for ensuring ecological survivability/stability in agricultural production systems [19]. A diverse gene pool across breeds and individuals protects the entire sheep sector against future challenges, including disease and extremes of climate. This study includes six breeds (Border Leicester, Norfolk Horn, Lincoln Longwool, Greyfaced Dartmoor, Oxford Down and Whiteface Woodland) that are included in the UK Breeds at Risk (BAR) list provided by the Department for Environment and Rural Affairs in the UK [20]. For a sheep breed to be included in the UK BAR list it has to be a native breed, with a population of less than 3000 breeding females, and as such would be considered to be at particular risk in the event of an outbreak of exotic disease [20].

Breeds that fall under the European Union’s definition of ‘rare’ are defined as having small and declining population sizes [21]. This can result in reduced genetic diversity and increased relatedness that manifests as inbreeding and inbreeding depression [22, 23]. Low diversity may result in a lack of genetic variation necessary to adapt to a changing climate or novel pathogen. Inbreeding is associated with the depression of a range of fitness and production-relevant traits in sheep breeds [24–27], including in rare breeds [28]. Moreover, some of the UK sheep breeds with small population sizes may be at additional risk from the deleterious effects of genetic drift, where alleles can become lost or fixed in a population, reducing overall breed fitness and increasing extinction risk [23]. Quantifying diversity and improving genetic-management of rare native sheep breeds in the UK is, therefore, critical.

Previous studies have used genotyping technology to examine genetic diversity within sheep populations from across the globe [29–31]. Despite their unique diversity and importance to global sheep production, native UK sheep breeds are poorly represented in these studies. The sheep HapMap study, which included 2,819 animals from 74 different breeds, demonstrated clear genetic separation between breeds globally, with notable genetic differences between sheep from diverse geographical origins [32]. Accordingly, this study aims to provide a preliminary dataset to characterise the genetic diversity of native UK sheep breeds with the objectives of, *i*) examining breed introgression, population structure and admixture, *ii*) quantifying within and between-breed genetic diversity and, *iii*) assessing the suitability of the Illumina 50 K genotyping chip for breed differentiation and its potential as a tool for genetic conservation purposes. Our hope is that this study will provide a foundation for a much larger characterisation of the genetic diversity represented by UK native sheep breeds.

## Materials and methods

### Sampling and genotyping

**Fig. 1:**
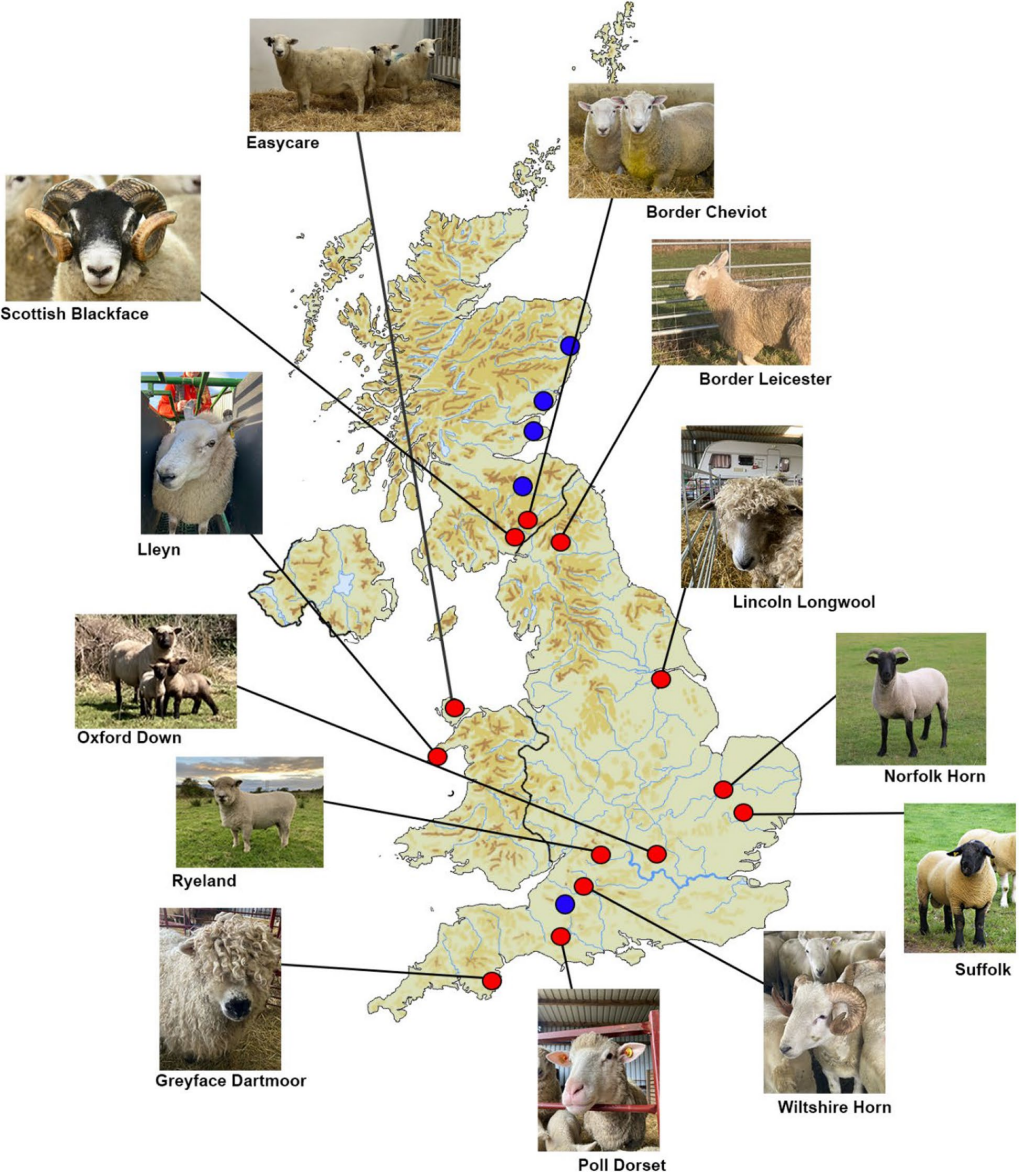
Geographic origins and sample localities of UK native breeds. Red points indicate breed origins, where-as blue points show sampling localities. The Wiltshire Horn was sampled from a location in the county of Wiltshire, all other sampling localities were in Scotland: Aberdeen, Perth, South Lanarkshire and Fife

From 2021 to 2023, nasal swab samples were obtained from 143 individual sheep from 13 different native breeds from four farms in Scotland and one farm in Wiltshire, England (Table 1, Table S1, Fig. 1). Six of these breeds appear on the UK BAR list and the Rare Breed Survival Trust (RBST) watchlist [14, 20]. One of these breeds, the Easycare^TM^, is a stable composite breed developed in the twentieth century with a large genetic contribution from the self-shedding Wiltshire Horn. Samples from each individual were collected and preserved using the PERFORMAgene PG-100 nasal swabs and stored at room temperature. DNA was extracted, by Neogen, according to the PERFORMAgene protocol and genotyped using the Illumina OvineSNP50 chip (Table S1). The Illumina 50 K Ovine SNP chip was selected as it has been widely used for other studies investigating genetic diversity in sheep populations, including for the sheep HapMap dataset [29, 30, 33]. The chip has a discovery panel of over 3000 sheep breeds, encompassing global ovine diversity, although native UK breeds are poorly represented [34].

**Table 1:**
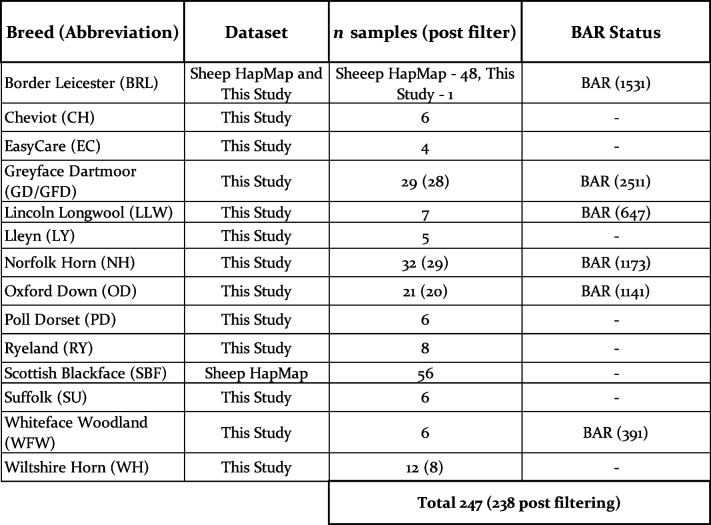
UK breeds and sample sizes included in this study. Number of individuals is given prior to, and post, filtering for call rate. The dataset was comprised of samples genotyped for this study and breeds added from the Sheep HapMap. Breeds included on the Breeds at Risk (BAR) list are indicated. Criteria for inclusion are an estimated breeding female population of under 3000 (accurate as of 03/2024) [20]

### Merging and filtering datasets

Samples collected for this study were genotyped at Neogen over a three-year period that covered the use of three chip versions, the OvineSNP50v2_HTS_20032166_A1 & A2, and OvineSNP50v3_HTS_20046264_B1.bpm (Table S1). Raw genotype data was converted to PLINK format using a custom script. Genotype data for two breeds, the Border Leicester and Scottish Blackface (Table 1), was extracted from the Sheep HapMap dataset. For all datasets, the BCFtools v.1 0.19 [35] plug-in ‘*fixref*’ was used to check, correct and fix strand orientation against the sheep reference genome version OAR3.1 (GCF_000298735.2). BCFtools *isec* was then used to create a list of shared SNPs between the four datasets (three different sampling occasions for this study plus the sheep HapMap dataset), before merging with BCFtools *merge*.

Merged data was filtered in PLINK 1.9 [36] on a SNP genotype call rate of > 90%, minimum individual call rate of 90%, and a minor allele frequency of 0.05 *(–geno 0.1, –mind 0.1, –maf 0.05*). Due to sample number heterogeneity between breed groups (Table 1), a pruned dataset was created where each group had between 4 and 8 individuals (Table S1, Table S2). To randomly select samples, each sample was assigned a randomly generated number, the list of samples was sorted and then the appropriate number of samples were chosen from the top of the sorted list. This resulted in 2 datasets: one with the study data merged with the HapMap data containing all samples, and one with the study and HapMap data merged and pruned for even sample sizes.

### Population structure and breed differentiation

Population structure and breed relationships were investigated by Principal Components Analysis (PCA), admixture, and a neighbour-net network. For PCA, admixture and the network analyses, datasets were pruned for linkage disequilibrium in PLINK 1.9 [36] (*–indep-pairwise* 50 10 0.2). PC analyses were calculated in PLINK 1.9 [36] from the covariance matrix (top 20 PCs). Admixture was performed for each dataset using ADMIXTURE [37], running from K = 2 to K = 20 and with 10 cross-validation in each set for error iterations. A neighbour-net network was constructed in Splitstree v.4.19.2 [38, 39] from a matrix of genetic distances generated from uncorrected p-distances.

### Relatedness, heterozygosity and inbreeding

The kinship coefficient was calculated for all pairs of sheep using relatedness2, based on the methods of Manichaikul et al. [40], and implemented within vcftools v.0.1.13 [41]. A custom script was used to format the output, before plotting in R v.4.0.0 [42] with the Heatmaply package [43]. Multi-locus heterozygosity was calculated as the proportion of sites at which an individual is heterozygous. Observed, O(HOM), and expected, E(HOM,) homozygosity was first calculated in PLINK 1.9 [36] with observed heterozygosity calculated as 1-(O(HOM)/n SNPs); Table S3). Inbreeding was inferred using runs of homozygosity (ROH) with the PLINK 1.9 [36] *–homozyg* function with the following parameters: minimum segment length of 1 MB *(–homozyg-kb 1000*), a minimum of 50 SNPs per segment (*–homozyg-snp 50*), a maximum of one heterozygous SNP (*–homozyg-het 1*) and a minimum SNP density of 70 kb per segment, (*–homozyg-density 70*). ROH results were reported as the percent of an individual’s genome in ROH, averaged by breed.

For breeds that had sample sizes > 10 prior to filtering (Border Leicester, Greyface Dartmoor, Norfolk Horn, Oxford Down, Scottish Blackface and Wiltshire Horn), demographic history was calculated by estimation of effective population size (*Ne*) using GONe [44], based on patterns of linkage disequilibrium (LD). Per-breed data was extracted from the merged dataset before filters, and filtered individually for missingness in PLINK 1.9 [36] *(–geno 0.1, –mind 0.1*). No maf or LD filters were applied as per the analysis recommendations.

## Results

### Assessment of genotyping data

We generated new 50 K genotype data for a total of 13 breeds and 143 individuals on the Illumina OvineSNP50 (Table 1, Table S1, Fig. 1). Autosomal genotyping rate varied from 99 to 44% per individual, with the majority of the dataset > 90%. Low call rates per individual were associated with DNA quality rather than breed. Merging of this data with the sheep UK breeds in the HapMap dataset added 48 samples for Border Leicester and 56 samples for Scottish Blackface (Table 1, Table S2). Post filtering (*–geno 0.1, –mind 0.1, maf 0.05*), the number of remaining SNPs was 35,217 in the full dataset and 35,247 in the pruned set. Nine individuals were removed due to low call rates, one Greyface Dartmoor, one Oxford Down, three Norfolk Horn and four Wiltshire Horn. The final number of breeds was 14, composed of 238 samples in the full set and 78 in the pruned set (Table 1, Table S1 & S2).

### Population structure: PCA, admixture and network analysis

**Fig. 2:**
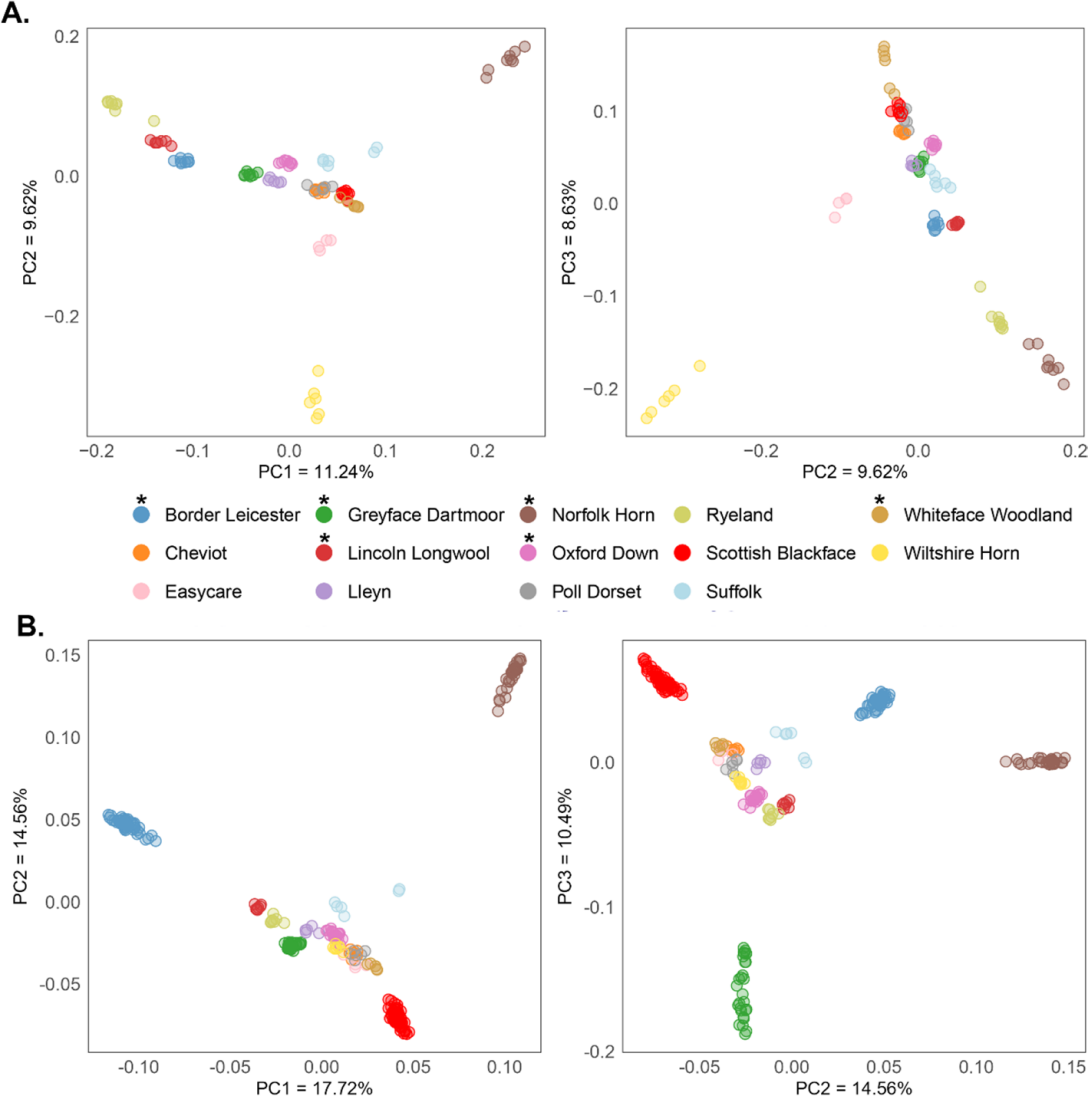
Principal components analysis (PCA) for UK sheep breeds. PCA (**A**) is shown for the dataset pruned for equal sample sizes (*n* between 4 and 8 per breed) and the full dataset is shown in (**B**). CH – Cheviot, LY – Lleyn, SU – Suffolk, EC- Easycare^TM^, WFW -Whiteface Woodland, SBF – Scottish Blackface, BL – Border Leicester, PD – Poll Dorset, GFD – Greyface Dartmoor, WH – Wiltshire Horn, OD – Oxford Down, RY – Ryeland, LLW – Lincoln Longwool, NH – Norfolk Horn. *Indicates breeds on the UK BAR list

Pruning the data for SNPs in high LD (r^2^ threshold = 0.2), reduced the number of SNPs to 22,000 in the full dataset and 13,960 SNPs in the set pruned for equal sample sizes. Principal components analysis showed a strong effect of sample size on the distribution of breeds in the PC space (Fig. 2A, B). Uneven sample sizes between groups can bias PCA analyses as larger groups disproportionately influence the mean and variance, skewing the PC’s and leading to larger groups artificially appearing more distinct [45]. This effect was obvious in our initial analysis of the whole dataset, with the breeds that have large sample sizes – the Greyface Dartmoor, Oxford Down and Norfolk Horn – all appearing distinct (Fig. 2B) while breeds with smaller sample sizes occupied a similar PC space to each other (Fig. 2A). Pruning the dataset so each breed has approximately even sample sizes (*n* = 4 to 8, minimum = 4, maximum = 8, Table S1), corrects for this effect (Fig. 2A).

Using the pruned dataset, PC1, PC2 and PC3 collectively accounted for 29% of the total genetic variance in the sheep. Component 1 has the Ryeland and the Norfolk Horn appearing distinct at either extremity of the axis, while PC2 separates out the Wiltshire Horn from all other breeds. Most rare breeds show some level of breed-distinctiveness while the Cheviot, Poll Dorset, Whiteface Woodland and Scottish Blackface occupy a similar PC space. The Lincoln Longwool and Border Leicester tend to group in close proximity to each other across PC’s, reflecting the historic role of the Longwool in the formation of the Border Leicester [46]. The Oxford Down, Greyface Dartmoor and Lleyn also tend to group together.

**Fig. 3:**
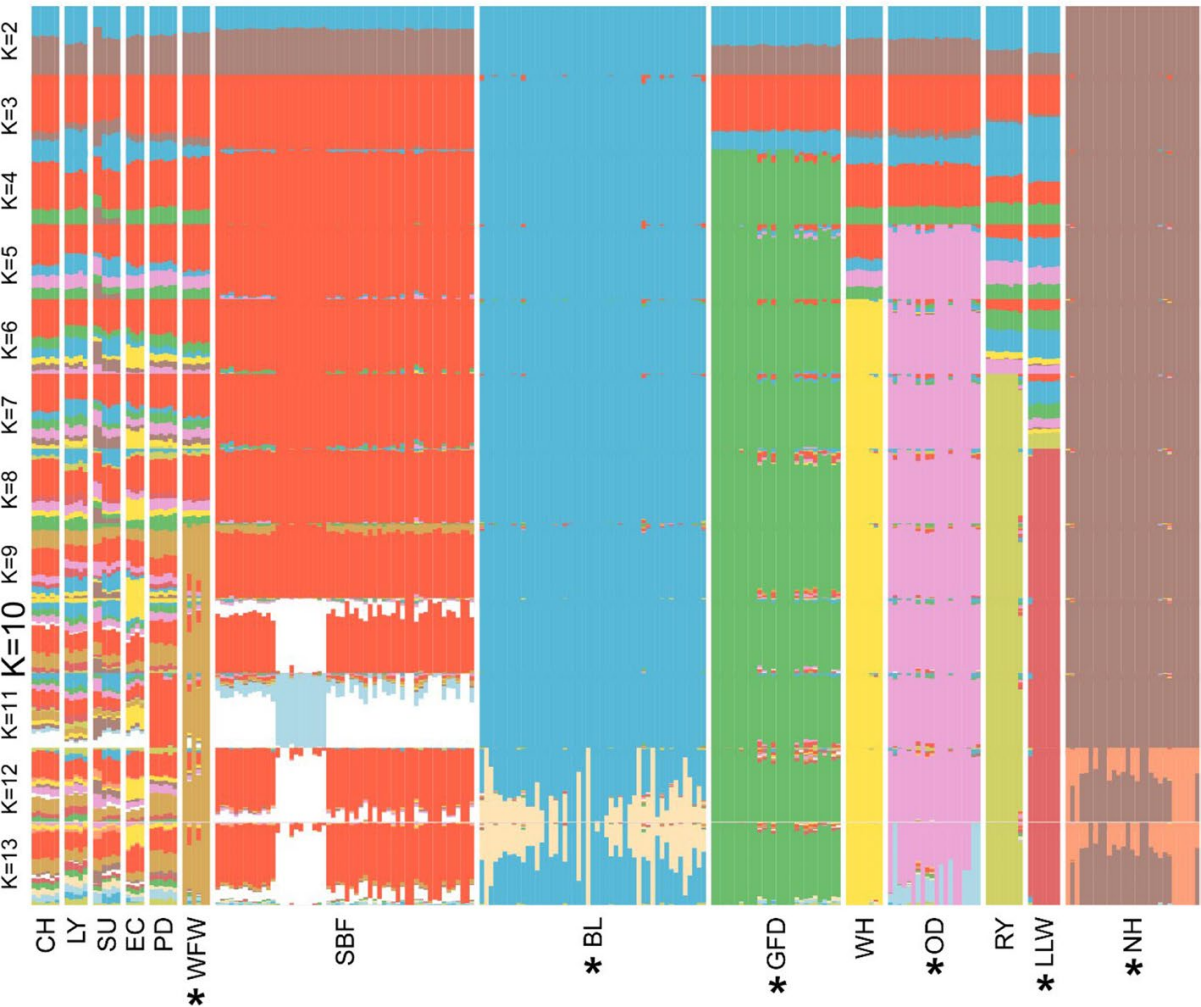
Admixture analyses for UK sheep breeds. The most likely value of K was K = 10. Production breeds tend to show higher admixture levels than rare breeds that show a high level of distinctiveness. CL – Cheviot, LY – Lleyn, SU – Suffolk, EC- Easycare^TM^, WFW -Whiteface Woodland, SBF – Scottish Blackface, BL – Border Leicester, PD – Poll Dorset, GFD – Greyface Dartmoor, WH – Wiltshire Horn, OD – Oxford Down, RY – Ryeland, SBF – Scottish Blackface, LLW – Lincoln Longwool, NH – Norfolk Horn. *Indicates breeds on the UK BAR list

Admixture was calculated for both the full (Fig. 3) and pruned (Figure S2) datasets, although the pruned data was not sufficient to fully capture breed complexity (Figure S2). The Border Leicester and Norfolk Horn show no admixture from K = 2 to K = 10. The Scottish Blackface emerges as a distinct entity as at K = 3, followed by the Greyface Dartmoor (K = 4), Oxford Down (K = 5), Wiltshire Horn (K = 6), Ryeland (K = 7) and Lincoln Longwool (K = 8). The admixed characteristics of the production breeds become apparent at K = 5*.* As the value of K increases after K = 10, within-breed ‘subtypes’ start to appear, most notably in the Scottish Blackface, Border Leicester, Oxford Down and Norfolk Horn. This is most pronounced from K = 12 where the analysis interprets these subtypes as distinct entities. The Poll Dorset breed shows no admixture at K = 11 despite being a highly admixed breed at most other values of K. Together with the emergence of subgroups, this suggests that K values higher than 10 may be over-fitted and not reflective of real biological groupings.

The most likely value of K for the dataset was K = 10 (cv error = 0.565; Figure S1). At this value of K, the commercial breeds—Cheviot, Lleyn, Suffolk, Easycare^TM^ and Poll Dorset show similar patterns of admixture and breed composition to each other and interpreted as a single breed by the analysis. However, the Easycare^TM^ has a notably higher contribution from the Wiltshire Horn than these other breeds, reflecting known genetic introgression from this breed in the creation of the Easycare^TM^ [47]. The remaining breeds show little to no admixture at K = 10, and the BAR-list breeds are notably distinct.

**Fig. 4:**
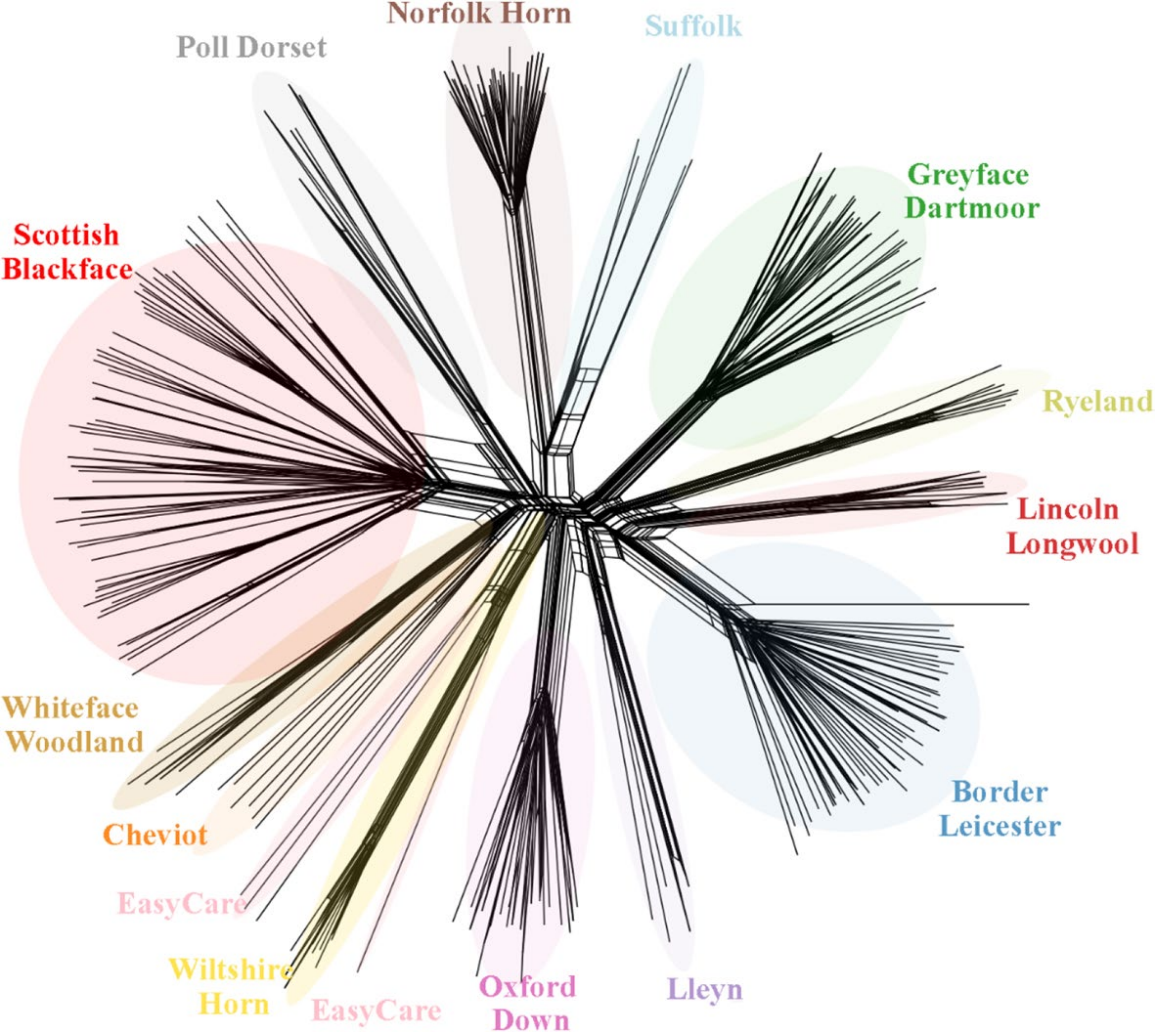
Splitstree neighbour-net network of native UK sheep breeds. The network is calculated from uncorrected p-distances on a dataset of 22,000 SNPs and 14 breeds. Some breed relationships not apparent in the PCA are observed here, for example the Suffolk and Norfolk Horn

The neighbour-net graph (Fig. 4) showed the same general pattern of increased distinctiveness of the rarer breeds compared to the commercial breeds that was apparent in the PCA and admixture analyses (Figs. 2 & 3). Several notable relationships are observed in the network, *i*) the Norfolk Horn and the Suffolk, *ii*) the Border Leicester, Lincoln Longwool and Ryeland, *iii*) the Poll Dorset and Scottish Blackface and, *iv*) the Easycare^TM^ and the Wiltshire Horn, with one Easycare^TM^ individual placing with the cluster of Wiltshire Horn branches. The Cheviot and Whiteface Woodland are somewhat undifferentiated from each other, and both are associated to the Scottish Blackface + Poll Dorset grouping.

### Demographic history

Reconstruction of effective population size (*Ne*) for six breeds (Border Leicester, Greyface Dartmoor, Norfolk Horn, Oxford Down, Scottish Blackface and Wiltshire Horn), showed that the BL, GFD, SBF and NH all have a reduced contemporary *Ne* compared to > 64 gens ago (Figure S3, where generation time is equal to 1 year). The OD and WH do not have large historical *Ne*, suggesting long-term effective population size depression. All breeds show a recent drop in *Ne,* but the production breeds exhibit more recent stability than the rare breeds. These results, however, are likely more reflective of individual flock history, rather than representative of breed history. More widespread sampling would have to be undertaken to achieve reliable demographic estimates for effective population size at the breed level.

### Kinship, heterozygosity and inbreeding

**Fig. 5:**
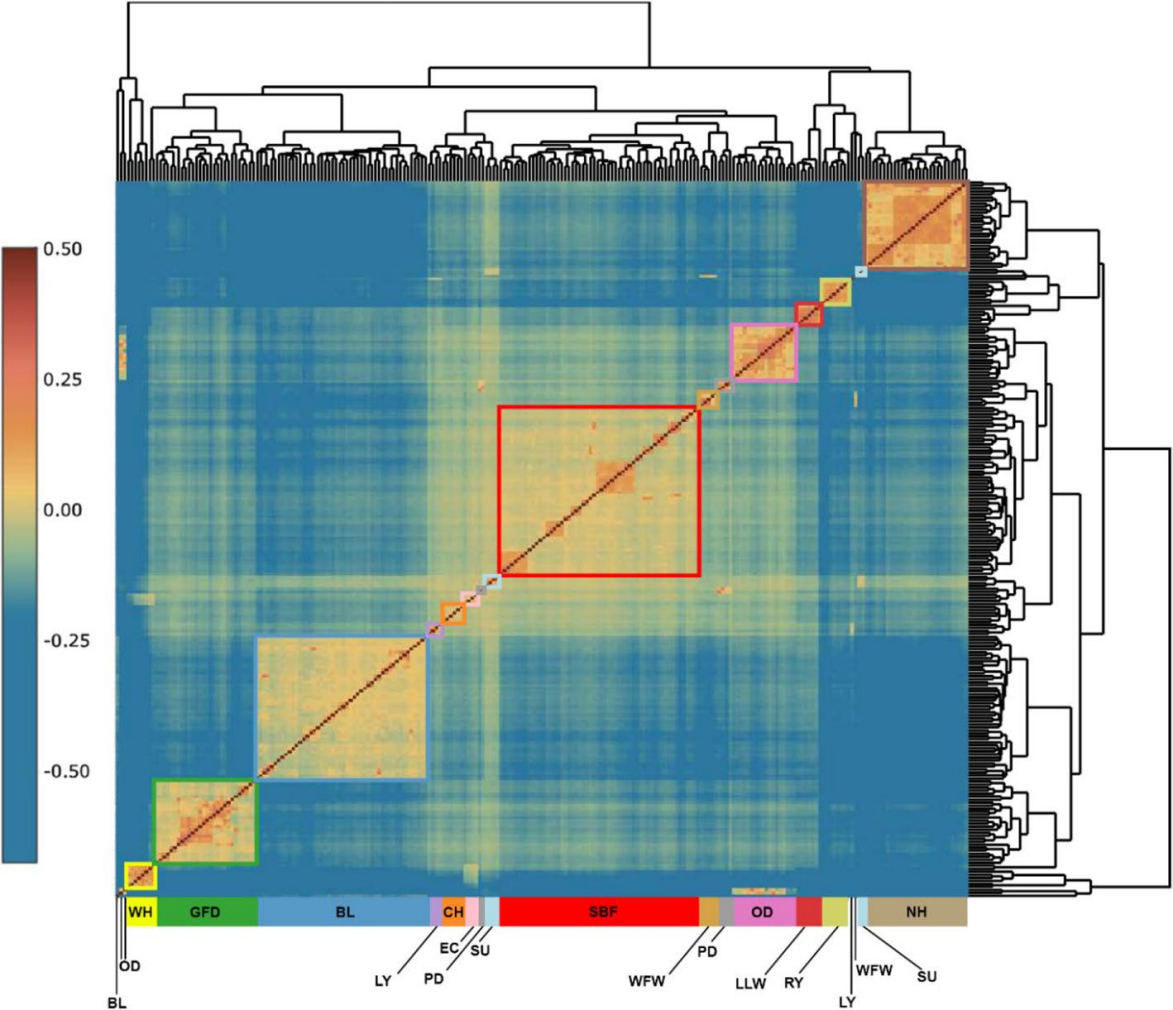
Pairwise kinship co-efficient for British sheep. A dendrogram based on kinship coefficient values from relateness2, reflecting breed boundaries and elevated within-breed relatedness for some groups. Colour scale based on relatedness categories where < 0.0884 = 3rd degree relations and below, 0.0884 – 0.177 = 2nd degree, 0.177 – 0.354 = 1st degree and > 0.345 = duplication/monozygotic twins. CH – Cheviot, LY – Lleyn, SU – Suffolk, EC- Easycare^TM^, WFW -Whiteface Woodland, BL – Border Leicester, SBF – Scottish Blackface, PD – Poll Dorset, GFD – Greyface Dartmoor, WH – Wiltshire Horn, OD – Oxford Down, RY – Ryeland, LLW – Lincoln Longwool, NH – Norfolk Horn

The relatedness analyses produced pairwise estimates of kinship coefficients for all pairs in the dataset; < 0.0884 indicates 3rd degree relatives, 0.0884 – 0.177 indicates 2nd degree relatives, 0.177 – 0.345 indicates 1st degree relatives and > 0.345 are duplicates/monozygotic twins. Negative values indicate the potential presence of structure between individuals and so were included in the analysis rather than transforming to zero. There were 288 pairs of 1st degree relations (parent-offspring, full siblings) present in the dataset (Table S4), which is reflective of the small population size of some of the breeds. The dendrogram produced by the Heatmaply package [43] (Fig. 5) shows the Wiltshire Horn as an outgroup with two large sister groups composed of (GFD, BL, LY, CH, EC, SU, SBF, WFW, PD and OD) and (LLW, RY, LY, WFW, SU, NH). The Suffolk was not monophyletic, with individuals placed in both sister groups, similar to the pattern seen in both the PCA (Fig. 2) and neighbour-net graph (Fig. 4), where two Suffolk individuals are slightly divergent from the other Suffolk samples.

The distinctiveness of each breed, based on relatedness, can be inferred from the within-to-outside kinship coefficient (orange to brown indicating higher levels of relatedness; blue to green indicating lower levels of relatedness). These relatedness patterns also show high congruity with the population structure analysis, with the Norfolk Horn, and Wiltshire Horn particularly well distinguished (Fig. 5). Notably, some of these breeds have pockets of high (1st degree) relatedness which can be observed as darker orange/brown areas within the breed. These are particularly apparent within the Scottish Blackface, Oxford Down and Norfolk Horn – four of the breeds which showed ‘subtypes’ in the admixture analysis at higher values of K (Fig. 3). The individuals showing elevated levels of kinship correspond to the individuals interpreted as sub-types in the admixture analysis, indicating that heterogeneity in relatedness within breeds is, at least in part, responsible for the observed patterns of admixture.

**Fig. 6:**
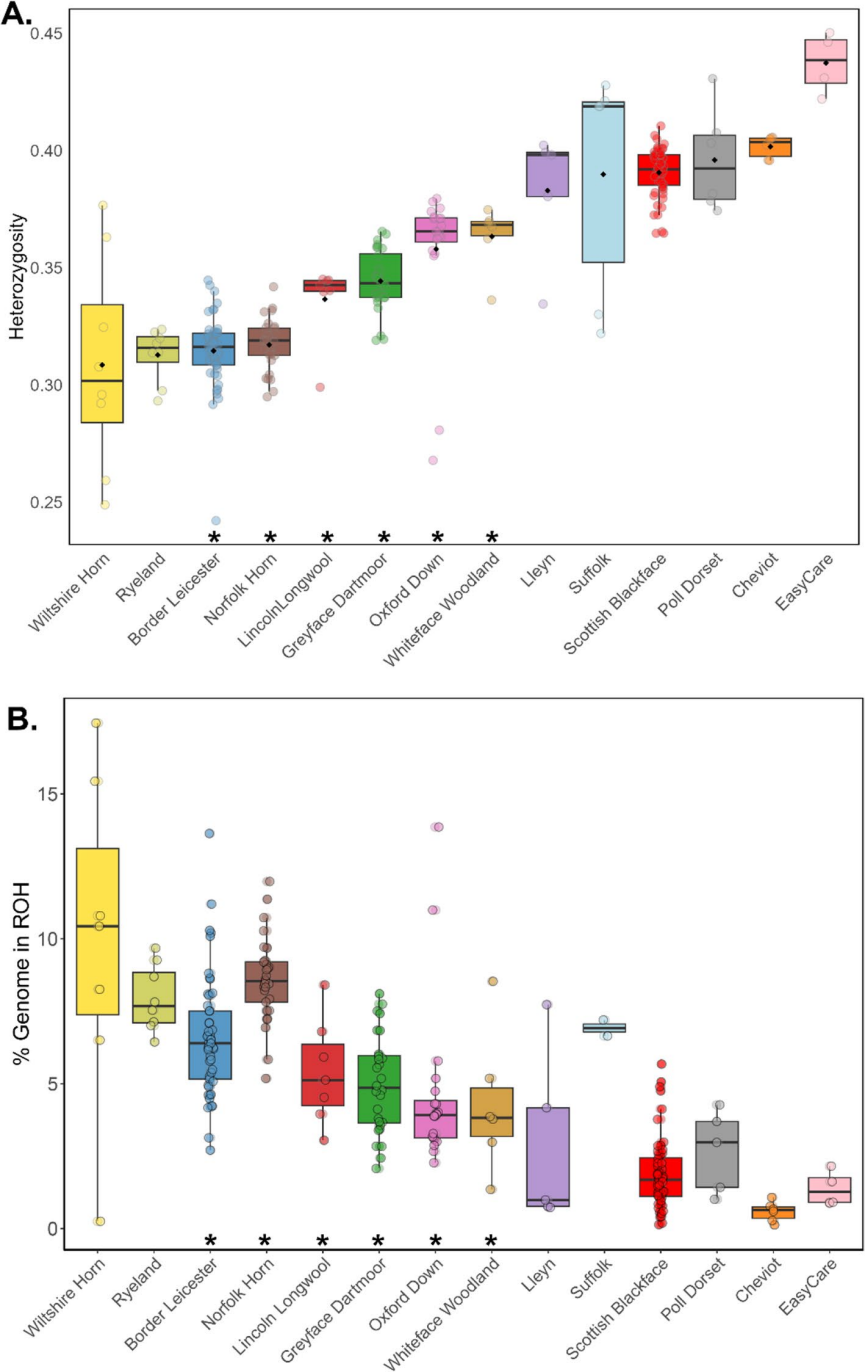
Heterozygosity (**A**) and runs of homozygosity (ROH, **B**) among native UK sheep breeds. Heterozygosity was calculated as the proportion of sites at which an individual is heterozygous after filtering for genotyping call rate and minor allele frequency. Asterix (*) indicates breeds on the UK BAR list. Both heterozygosity and ROH are shown by individual and averaged by breed (black bar)

Despite the apparent elevated kinship levels, heterozygosity was within the range reported in the literature for sheep [29]. Mean heterozygosity per breed ranged from a min of 0.3 in the Wiltshire Horn (but with a large range of 0.3 to 0.38) to 0.44 in the Easycare^TM^ (Fig. 6A). While sample size may influence these results, heterozygosity was clearly higher in the Cheviot, Lleyn, Suffolk, Easycare^TM^, Scottish Blackface and Poll Dorset - all breeds which have large population sizes. Breeds on the BAR list all show the lowest heterozygosity in the dataset, but the lowest diversity was observed in two breeds not listed on the BAR – the Wiltshire Horn and the Ryeland. Conversely, the Oxford Down and the Whiteface Woodland, both BAR listed breeds, show reasonably good levels of mean heterozygosity, although two Oxford Down samples were low outliers.

The percent of the genome covered by runs of homozygosity (ROH) mirrored heterozygosity almost exactly, with more heterozygous breeds/individuals showing smaller proportions of their genomes in ROH than those that have less genetic diversity (Fig. 6B, Table S5). The Wiltshire Horn, Norfolk Horn and Ryeland had the highest means for this metric but there was a huge range in %ROH within the Wiltshire Horn; one individual showed an almost complete absence of ROH while another had the most %ROH in the dataset at > 15%. While the Suffolk also appears to have a high %ROH, this is on the basis of only two inbred individuals as the other samples in the breed did not show any ROH that met the analysis criteria and so were not included in the results. There is also considerable within-breed variation in some cases, for example within the Oxford Down, Border Leicester, Wiltshire Horn and Lleyn.

## Discussion

Our results represent the first study to systematically examine the genetic diversity and breed relationships of UK native sheep breeds. They highlight intriguing, and previously unknown, genetic profiles. For example, the breeds associated with larger population size and production use such as the Easycare^TM^, Cheviot, Suffolk and Lleyn consistently show increased admixture and levels of genetic diversity, exhibit low degrees of genetic differentiation and have the lowest kinship co-efficients. These findings are unsurprising considering that these breeds are widely used in commercial sheep production in the UK. The strong genetic similarity among them may be attributed to shared breeding objectives that prioritise specific production relevant traits or the historical breeding relationships. Additionally, these breeds are primarily bred for economic productivity rather than for rare breed status. This means breeding practices within these breeds are likely to have a reduced emphasis on rigorous pedigree monitoring to maintain purebred animals and more crossbreeding with other commercial breeds.

In contrast, some of the less numerous native breeds demonstrated pronounced genetic differentiation, low levels of admixture, genetic diversity and higher inbreeding and within-breed relatedness. Our analysis has shown the genetics of the Wiltshire Horn to be particularly unique relative to the other breeds we investigated. The Wiltshire Horn breed is not on the UK BAR list but is considered relatively rare due to its limited population of fewer than 15,000 ewes [48]. It was previously on the RBST watchlist. Our genetic diversity analysis consistently demonstrates that the breed forms a unique and isolated group, distinct from other breeds included in the study. Also, the breed exhibits no detectable admixture with other breeds across a range of estimated ancestral populations which strongly suggests that the Wiltshire Horn originated from a unique ancestral population. This is possibly attributable to the very diligent maintenance of genetic integrity in the breed by a handful of passionate breeders as well as the early implementation of thorough pedigree monitoring practices within the breed (Society). However, this breed also showed the lowest heterozygosity of all breeds in the study and high within-breed relatedness.

In both the PCA and neighbour-net graph the Border Leicester and Lincoln Longwool appeared to share a close genetic relationship. These are both ‘Longwool’ breeds, which are some of the oldest documented UK breeds, with their ancestral types dating back to at least the Middle Ages (c. 500 to 1500 AD) [46]. English Longwool rams were major founders of many contemporary British breeds, and both the Lincoln Longwool and the Border Leicester have the English Longwool as one of their founding varieties [46]. The proximity of these breeds to the Ryeland is surprising, given that the Ryeland is an ancient short-wool breed, that has remained distinct. However, there is some suggestion that the noted eighteenth century breeder Robert Bakewell [49], folded the Border Leicester into the Ryeland [50], which may explain this result and also highlights the utility of genotyping to uncover poorly documented breed histories.

Interestingly the Norfolk Horn has maintained considerable genetic distinctiveness, even in the wake of historical backcrossing efforts with Suffolk sheep [51]. Notably, these two breeds do not coalesce into a single group in the PCA space but the neighbour-net graph highlighted their historical relationship (Figs. 2A, 3). Throughout the twentieth century, Norfolk Horns faced a decline in popularity, and by the First World War, only one flock of the breed remained [51]. These surviving Norfolk Horns were characterised by extreme levels of inbreeding, rendering any attempts to expand the flock seemingly insurmountable. To tackle this, a back-crossing program using Suffolk sheep was initiated. The program spanned several decades. Today, there are over 2,500 Norfolk Horns in existence [52]. This compelling evidence attests to the efficacy of the back-crossing program in rescuing the Norfolk Horn species while safeguarding the integrity of their unique genetic heritage. It serves as a resounding testament that well-planned and persistent interventions can combat the challenges posed by inbreeding.

Our examination of admixture in each of the breeds importantly showed that several of the breeds with increased admixture are the more numerous and commercially utilised breeds. For example, the Easycare^TM^, Cheviot, Lleyn and Suffolk breeds show more admixture than the Wiltshire Horn, Lincoln Longwool, Ryeland and Whiteface Woodland, all of which show no admixture. This observation aligns with the notion that these commercial breeds are likely to have been selectively bred for desirable commercial traits. The increased levels of admixture in these breeds are significant as it implies a greater genetic diversity within the breeds. This means they may have increased breed resilience and adaptability than the rarer breeds with lower levels of admixture. The low admixture in the rarer breeds underscores the importance of protecting these breeds from the negative consequences of inbreeding. Doing so is critical for preserving genetic heritage and breed purity.

The mean heterozygosity values recorded in this study (0.3 in the Wiltshire Horn to 0.44 in the Easycare^TM^) were similar to those observed for UK and European breeds in other studies e.g., Kijas et al. [29] who reported a range of 0.29–0.32. However, for some of the native UK breeds studied, including those listed on the UK BAR list, there are indications of potential inbreeding occurring, which could pose a significant threat to the long-term survival of these breeds. The Wiltshire Horn has the lowest value of heterozygosity of the sheep studied and highest average percentage of the genome in ROH (but with high within-breed variability). This is possibly due the small number of ewes that are registered annually for the breed [48] which can result in a smaller gene pool. Given the unique sustainable traits of the Wiltshire Horn, such as seasonal wool shedding, and its clear genetic distinctiveness from other breeds studied, this is particularly concerning.

The Norfolk Horn, another breed with unique traits and clear genetic distinctiveness from other breeds studied, also has particularly low values of heterozygosity. This is perhaps explainable by its historical bottle-neck in the 1970’s and subsequent rescue via crossing with the closely related Suffolk [51, 52]. More generally, the trend is that the breeds on the UK BAR list, with smaller population sizes, appear to have lower levels of heterozygosity and subsequent greater degrees of inbreeding. Inbreeding elevates the risk of individuals inheriting two copies of recessive deleterious genes, which can have adverse effects, including reduced fertility [22]. Considering the potential consequences, it is imperative to address the challenge of inbreeding to ensure breed preservation. Early detection of excessive inbreeding using genotyping tools can facilitate the implementation of well-designed breeding and cross-breeding programs, perhaps including importation of novel genetics from European flocks. Both the Oxford Down and the Whiteface Woodland, two BAR listed breeds, showed moderate-good levels of genetic diversity and (with the exception of a small number of outliers), low inbreeding. This demonstrates that small population size need not inevitably result in poor genetic diversity, as long as management strategies are sufficient to combat the effects of small numbers of breeding individuals. The findings of this study demonstrate that some degree of routine genotyping and comprehensive recording of easy to measure traits would be warranted, particularly for the UK breeds on the BAR list.

The sample sizes between breeds in this study were variable with most breeds including between 4 and 8 individuals and four breeds with sample sizes > 20. In addition, sampling was limited in some cases to single flocks that may or may not be typical or representative of the breed population as a whole. This reflects the sampling limitations of the study and affects statistical interpretation, most obviously by skewing the mean and variance (for example, in PCA space (Fig. 2) and in heterozygosity estimates (Fig. 6A)). Future research would benefit from increased per-breed sample sizes and more widespread pedigree informed sampling of flocks, including examples of graded-up individuals and flocks with a history of introgression from imported individuals.

This study represents an essential first step in the ongoing pursuit for more comprehensive genetic characterisation of native UK sheep breeds. Further research should obtain larger sample sizes for each breed and include several different flocks and geographical locations within the UK. Whole-genome sequencing, using short read Illumina data, would provide many million more SNPs for analysis and more accurate quantification of genetic diversity in UK native sheep breeds as well as identification of genetic markers driving traits of interest for breed improvement. From the whole genome sequencing data examination of runs of homozygosity and structural variants could also help to define the degree of inbreeding [53]. Whole genome sequencing could also be used to validate the content on existing SNP chips or design new tools with content tailored specifically for UK sheep breeds, enhancing the precision of SNP genotyping and informing future breeding and cross-breeding programmes. Furthermore, global pangenome efforts for sheep to characterise and conserve global genomic diversity using long read genome sequencing technologies [54] should include the UK native breeds [6].

## Conclusions

This study emphasises the importance of conserving the native UK sheep breeds due to their unique genetic diversity. These breeds provide important and irreplaceable genetic resources, that will be essential for the UK sheep sector in decades to come as climate and other pressures on production increase.

## Supplementary Information

Additional file 1: SI_Figures_UKsheep. Supplementary Figures.

Additional file 2: SI_Tables_UKsheep. Supplementary Tables.

## Data Availability

The datasets generated and/or analysed during the current study (including code) are available in the Dryad Digital Repository: https://doi.org/10.5061/dryad.z08kprrnm and the sheep HapMap https://sheephapmap.org.
