# Supplementary material for: Analysis of genotyping data reveals the unique genetic diversity represented by the breeds of sheep native to the United Kingdom
Source: BMC Genom Data. 2024 Sep 17;25:82. doi: 10.1186/s12863-024-01265-3 (PMC11409796; doi:10.1186/s12863-024-01265-3)
Supplement: Supplementary file 1 — Additional file 1: SI_Figures_UKsheep. Supplementary Figures. [file 12863_2024_1265_MOESM1_ESM.docx]

**Analysis of genotyping data reveals the unique genetic diversity represented by the breeds of sheep native to the United Kingdom**

Eleanor Kerr^1^, Melissa M. Marr^1^, Lauren Collins^1^, Katie Dubarry^1^, Mazdak Salavati^2^, Alissa Scinto^1^, Shernae Woolley^1^, Emily Clark^1^

^1^The Roslin Institute, University of Edinburgh, Easter Bush Campus, Easter Bush, Midlothian, EH25 9RG;
^2^Scotland’s Rural College (SRUC), Dairy Research Centre, Barony Campus, Dumfries, DG1 3NE

^*^Correspondence [emily.clark@roslin.ed.ac.uk](mailto:emily.clark@roslin.ed.ac.uk)

**Supplementary Figures**


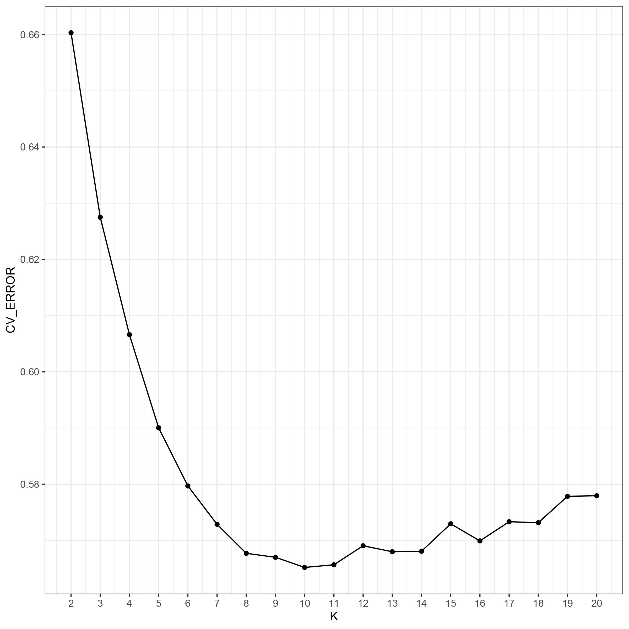


**Figure S1. Cross-validation errors for admixture analysis.** Ten CV iterations were performed, most likely value of K is 10 (cv = 0.565)


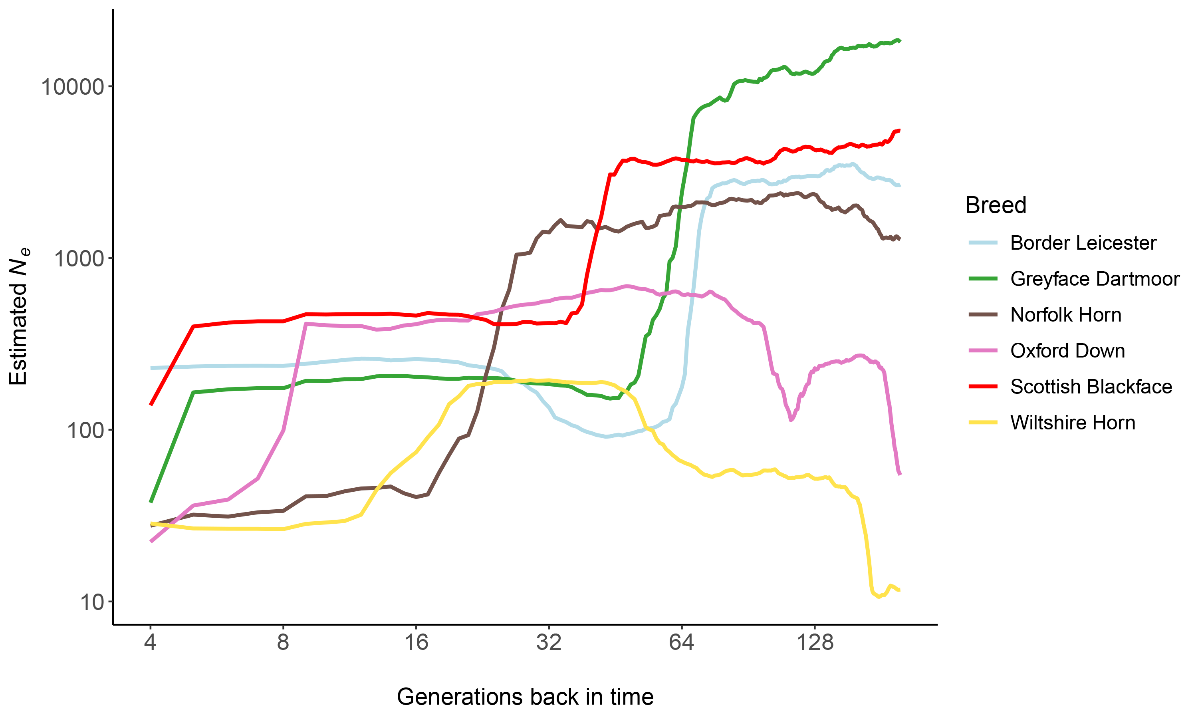


**Figure S2. Effective population size for UK sheep**. Performed on c. 30k SNPs per breed group with one generation equal to one calendar year. Production breeds generally show a larger and more stable *Ne* than rare breeds but this is likely affected by limited flock sampling within breeds.
